# A Narrative Review of Ethical Issues in Precision Psychiatry: Mapping Unresolved Tensions Across Modalities

**DOI:** 10.3390/jpm16070337

**Published:** 2026-06-23

**Authors:** Christos Doukas, Petros Galanis, Athanasios Douzenis, Panagiota Bali, Marie Louise Psarra, Ioannis Michopoulos, Nikolaos Smyrnis, Konstantinos Tasios

**Affiliations:** 1 Second Department of Psychiatry, Attikon University Hospital, School of Medicine, National and Kapodistrian University of Athens, 1 Rimini St., 12462 Athens, Greece; cristdoukas@gmail.com (C.D.); thandouz@med.uoa.gr (A.D.); pabali@med.uoa.gr (P.B.); mlpsarra@yahoo.gr (M.L.P.); imihopou@med.uoa.gr (I.M.); ktasios@med.uoa.gr (K.T.); 2 Epidemiology and Public Health, Faculty of Nursing, National and Kapodistrian University of Athens, 123 Papadiamantopoulou St., 11527 Athens, Greece; pegalan@nurs.uoa.gr

**Keywords:** precision psychiatry, personalised psychiatry, ethics, bioethics, ethical issues, ethical tensions, digital phenotyping, explainability, equity

## Abstract

Precision psychiatry promises a more objective and effective approach to psychiatric care, yet its implementation raises growing ethical challenges as technology advances. This narrative review offers a qualitative synthesis of the ethical issues reported in 62 studies, with emphasis on the practical tensions that arise when core principles conflict. Rather than organising concerns around traditional ethical principles, the review maps them across the main modalities of precision psychiatry, namely genomics, neuroimaging, digital phenotyping, and AI-driven interventions. Four explicit positions are advanced. First, equity must be engineered from the outset rather than assumed. Second, interpretability should outweigh marginal gains in accuracy in a field built on subjective report. Third, stigma is bidirectional and contingent on framing and the availability of meaningful intervention. Fourth, individualised care must demonstrate clinical and economic superiority over standardised approaches. Precision psychiatry is likely to reshape psychiatric practice and the therapeutic relationship itself. Interdisciplinary collaboration, clear guidelines, and continuous ethical vigilance will be essential for responsible adoption and sustained public trust.

## 1. Introduction

Psychiatry has always been a personalised specialty. It rests on descriptive psychopathology and phenomenology, both of which involve subjective interpretation [1]. That subjectivity, together with the absence of objective assessment tools, can weaken the accuracy and effectiveness of clinical evaluation [2,3,4]. Precision medicine takes a different route. It draws on individual characteristics, from genomic data to environmental exposures, to build and test personalised clinical prediction models for screening, diagnosis, prognosis, and treatment stratification [2,5,6].

The past century saw real progress in psychiatric pharmacology and in public awareness of mental health. Yet the expansion of therapeutic options has felt slower and less consistent than in other branches of medicine [1,7]. Psychiatric science has long stressed an individualised approach to treatment, but most of its data came from studies on patient groups defined by standard diagnostic criteria [3]. Diagnostic ambiguity and therapeutic uncertainty remain common features of the field [8]. A turning point came when researchers linked the genome to drug metabolism, which opened the door to individual predictions about treatment effects and helped precision psychiatry take shape [1,7,9]. Advances in genetic sequencing, non-invasive neuroimaging, and machine learning have multiplied the information available to clinicians [3,10,11]. Over the past decade, many clinical prediction models have been built to identify people at high risk for mental disorders, sharpen diagnosis, and improve treatment response [12].

The growth of precision psychiatry runs into several obstacles. The most visible are ethical, and they can slow both clinicians and policymakers in taking up the new tools [2,3]. These concerns stem from questions about therapeutic equity, scientific validity, and cost–benefit balance [1,13]. Psychiatry also carries features that sharpen the problem: the compromised self-determination of many patients and the deep-rooted social attitudes toward mental illness [2]. Newer worries concern artificial intelligence (AI) and how data are handled, including limits on transparency, accuracy, and bias in model outputs [10,12]. Five ethical principles usually guide the evaluation of psychiatric precision: autonomy, beneficence, non-maleficence, justice, and interpretability [2]. But the principles can conflict, and prioritising them is rarely straightforward [2,14]. Assessing safety and efficacy is necessary, yet delays driven by ethical or cultural debate carry their own costs, because untreated disorders progress and prognosis worsens [3,15]. Mental health promotion matters for patient rights and quality of life. Given the rising interest in emerging psychiatric models and the growing body of recent literature [2,5,6,10], this review aims to synthesise evidence on the ethical dimensions of precision psychiatry and to inform the future development of ethical frameworks and strategies addressing associated challenges, ultimately facilitating broader participation and practical application.

The ethical dimensions of precision psychiatry have already drawn substantial commentary, and the field does not lack catalogues of concern [2,3,12]. What it lacks is resolution. Previous reviews catalogue the risks but rarely adjudicate between them, so the points at which one ethical commitment is bought at the expense of another remain largely unexamined. The result is guidance that affirms every principle at once and leaves the clinician without a way to act when two of them collide. Existing reviews tend to list principles and risks, yet several of these pull against one another in ways that remain unaddressed. Equity is upheld as a goal, while the patients with the most severe illness are also the most likely to opt out, so the technology may widen the gap it promises to close. Interpretability is urged, yet the accuracy-first trade-off borrowed from other branches of medicine sits poorly in a discipline whose diagnoses rest on subjective report. Biological framing is said to reduce blame, and in the same literature, it is said to deepen determinism and exclusion. Personalised care is the stated aim, while the economics of the field still reward standardised products for broad markets. This review is organised around these tensions rather than around a fixed set of principles. It maps the ethical questions across the main modalities now in use, from genomic and neuroimaging tools to digital phenotyping and conversational agents. Each concern is tied to the technology and the application that produce it. Emerging areas, including screening in minors, prenatal testing, and forensic use, are brought into one frame. The aim is to show where the literature remains unresolved and to set out the authors’ reading of how the tensions might be approached.

## 2. Materials and Methods

This study constitutes a narrative review of the literature examining the ethical implications of precision psychiatry. A narrative design was chosen because the aim was to map and interpret a broad, conceptually diverse body of work, rather than to estimate a pooled quantitative effect, which is the purpose better served by a systematic review. Relevant publications were identified through structured searches conducted in the electronic databases PubMed (National Library of Medicine, Bethesda, MD, USA) and Google Scholar (Google LLC, Mountain View, CA, USA), between 10 January and 21 March 2026. No restriction was placed on publication date; the majority of the included literature was published between 2020 and 2026, with a small number of foundational earlier works retained for conceptual grounding. The search strategy incorporated combinations of the following keywords: “precision psychiatry”, “personalized psychiatry”, ethics, “ethical issues”, bioethics, “informed consent”, privacy, stigma, and discrimination.

**Figure 1:**
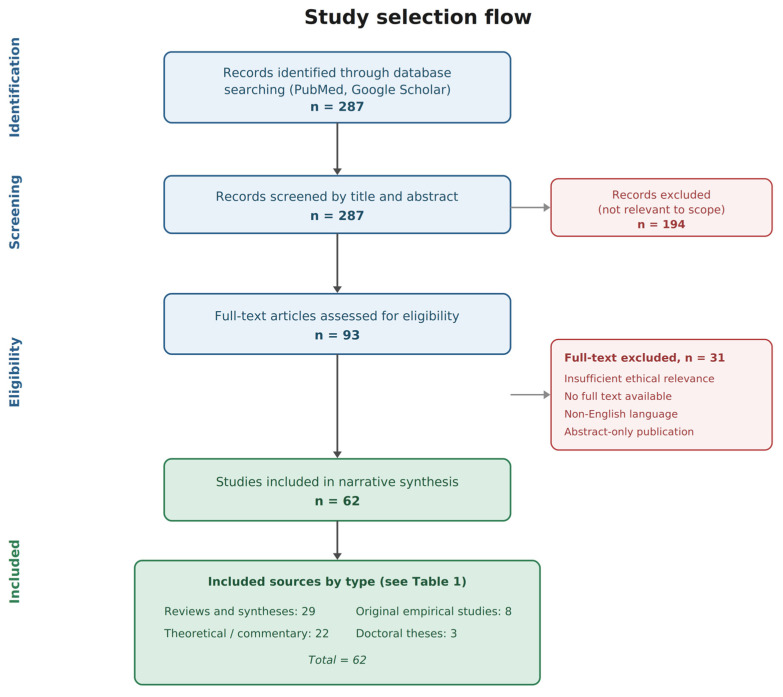
Flow of study selection. Of 287 records identified in PubMed and Google Scholar, 194 were excluded at title and abstract screening and 31 at full-text assessment, leaving 62 studies for narrative synthesis. The composition of the included sources by type is reported in Table S1.

The initial search yielded a total of 287 records. Following title and abstract screening, 194 records were excluded due to lack of relevance to the scope of the review. The remaining 93 articles were retrieved for full-text assessment. After full-text evaluation, 31 articles were excluded based on predefined criteria, including insufficient relevance to ethical considerations, lack of full-text availability, non-English language, or classification as abstract-only publications. A total of 62 studies were ultimately included in the present narrative synthesis. Study selection and data evaluation were primarily conducted by one reviewer, with consultation from a second reviewer in cases of uncertainty, in order to enhance consistency and minimise selection bias. Figure 1 summarises the selection process.

In keeping with established guidance on narrative synthesis, a formal risk-of-bias or quality-grading instrument was not applied, since the objective was conceptual mapping rather than effect estimation. To make the character of the evidence base explicit, the included sources were classified by type (Table S1): 29 reviews and syntheses, 22 theoretical, conceptual, or commentary articles, 8 original empirical studies, and 3 doctoral theses. Reviews and conceptual work predominated, and original empirical studies were comparatively few. This distribution reflects the youth of the field and the volume of synthetic and theoretical writing it has so far generated, and it is one reason an interpretive synthesis that states the open tensions plainly is useful at this stage. The limitations of this approach, including the single primary screener and the use of two databases, are addressed in Section 4.3.

This review is organised into thematic sections derived from the identification of recurrent patterns and conceptual domains across the included studies. These themes emerged through iterative analysis and qualitative synthesis of the selected papers, with emphasis placed on the most salient ethical challenges and considerations associated with the application of precision psychiatry.

## 3. Relevant Sections

**Figure 2:**
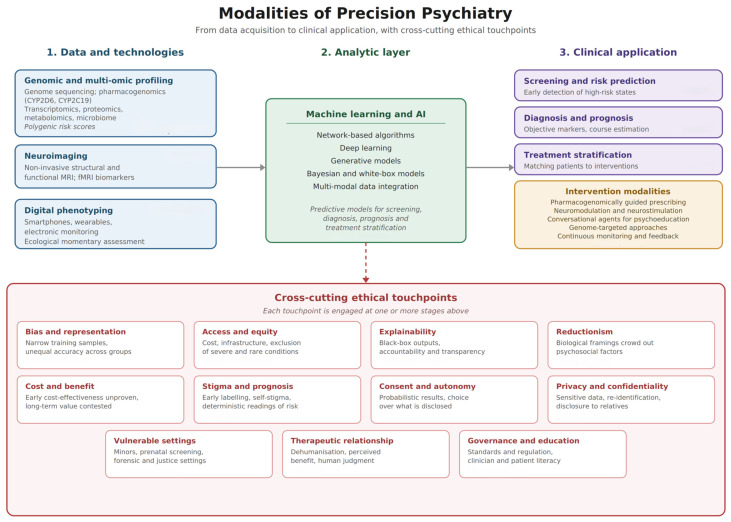
Modalities of precision psychiatry. The diagram reads left to right across three stages. Data and technologies (genomic and multi-omic profiling [8,9,12,16,17], neuroimaging [3,11,18,19], and digital phenotyping [20,21,22,23,24]) feed an analytic layer of machine learning and artificial intelligence [10,11,16,23,25], which informs clinical applications (screening [5,12,26], diagnosis and prognosis [2,6,14,25], and treatment stratification [5,8,14]) and the interventions that follow. The lower panel lists the cross-cutting ethical touchpoints discussed in this review; bias and representation [10,12,16,21,23,30], access and equity [10,20,28,30,31,32], explainability [10,11,19,23,33,34], reductionism [8,12,35,36,37], cost and benefit [13,32], stigma and prognosis [5,12,15,30,38], consent and autonomy [10,12,37], privacy and confidentiality [21,23,27,39], vulnerable settings [6,12,25,29,40,41], therapeutic relationship [2,14,27], governance and education [12,19,20,42,43]. Each of these ethical dimensions is engaged at one or more stages of the framework, rather than being associated with a single modality.

Precision psychiatry draws on several distinct modalities, grouped in Figure 2 into three stages. The first stage covers data and the technologies that generate it. Genomic and multi-omic profiling supplies sequence data and pharmacogenomic markers such as CYP2D6 and CYP2C19 and extends to transcriptomic, proteomic, metabolomic, and microbiome layers that feed polygenic risk scores [8,9,12,16,17]. Non-invasive neuroimaging adds structural and functional measures of the brain, including fMRI biomarkers [3,11,18,19]. Digital phenotyping captures behaviour in daily life through smartphones, wearables, electronic monitoring, and ecological momentary assessment [20,21,22,23,24]. The second stage is the analytic layer. Here machine learning and artificial intelligence integrate heterogeneous inputs through network-based algorithms, deep learning, generative models, and Bayesian and white-box models, with multi-modal data integration across sources [10,11,16,23,25]. These methods build the predictive models that drive the third stage. In care, the outputs support screening and risk prediction [5,12,26], diagnosis and prognosis [2,6,14,25], and treatment stratification that matches patients to interventions [5,8,14]. The interventions themselves span pharmacogenomically guided prescribing, neuromodulation and neurostimulation, conversational agents for psychoeducation, genome-targeted approaches, and continuous monitoring and feedback [9,13,27,28,29]. Each modality raises ethical questions, and these recur across stages rather than belonging to any single one. The lower panel of Figure 2 sets out the cross-cutting touchpoints examined in this review: bias and representation, where narrow samples yield uneven accuracy (Section 3.2.1); access and equity, shaped by cost and exclusion (Section 3.2.2); explainability, covering black-box outputs and accountability (Section 3.2.4); reductionism, where biological framings crowd out psychosocial factors (Section 3.2.3); cost and benefit, where early cost-effectiveness stays unproven (Section 3.2.5); stigma and prognosis, from early labelling and self-stigma (Section 3.2.8); consent and autonomy, given probabilistic results and disclosure choices (Section 3.2.9); privacy and confidentiality, against re-identification of sensitive data (Section 3.2.10); vulnerable settings in minors, prenatal, and forensic contexts (Section 3.2.14, Section 3.2.15 and Section 3.2.16); the therapeutic relationship, where dehumanisation meets human judgement (Section 3.2.6); and governance and education, covering standards and clinician literacy (Section 3.3, Section 3.2.12 and Section 3.2.13).

### 3.1. Advantages

Treatment stratification matches patients with the interventions best suited to them, so that resources go where they are most likely to help and fewer people receive unnecessary or ineffective therapies [5]. Early detection of mental disorders through biomarker-based screening can reduce risk or at least delay onset and progression [12,26]. Close monitoring of people at high risk, paired with early intervention, may lower severity and chronicity, cut socioeconomic costs, and improve clinical outcomes, with a positive effect on quality of life [12,14,44]. Prognostic information also gives patients a clearer picture of what they face, encourages behavioural changes that address modifiable risk factors, and supports longer-term planning [12,45]. Knowing one’s individual risk can bring a sense of control [12]. Patients themselves report that insight into brain function and measurement profiles renews their motivation to engage with treatment and helps gradually reduce stigma [46].

Sharing predictive genetic test results can also strengthen family relationships and help relatives prepare for the care of at-risk members [12]. The use of such models pushes back against paternalism. Patients gain more say in what information they receive and in what they choose to do with it [12]. Poor treatment optimisation in mental illness leads to weaker responses, more adverse effects, and lower compliance [20]. Physicians carry a moral duty to provide the best care available, and precision psychiatry may help them meet that duty, particularly in chronic and severe disorders such as psychotic illnesses, where functional impairment runs deep and course varies widely [20]. Predicting who will respond to which treatment remains hard. But progress in precision medicine is expected to shift that picture [8,14]. Better stratification and greater pharmacological specificity should also feed back into future drug development [8].

### 3.2. Disadvantages—Areas of Concern

#### 3.2.1. Risk of Bias and Unequal Representation

Polygenic risk scores for mental disorders should be built from diverse samples that represent the relevant population [8,12]. In practice, many mental health models draw on majority populations or on convenience and voluntary samples [10]. The overrepresentation of developed countries, together with machine learning algorithms trained on narrow datasets, can entrench existing inequalities and biases, racial and gender bias among them. Predictive accuracy suffers for minority, marginalised, vulnerable, underdiagnosed, or misclassified groups, which in turn creates diagnostic and treatment gaps and can harm patients who are already underrepresented [4,11,12,16,18,19,23,25,27,33,47,48,49,50,51,52,53,54]. For instance, a depression risk predictor designed for young adults may systematically underestimate risk in older adults because the data supporting the relevant associations are sparse [23]. Clinical consequences also appear in cases of genetic variation between ethnic groups, such as differences in CYP2D6 and CYP2C19 cytochromes [9]. Evidence suggests that lower educational attainment, male gender, and co-occurring anxiety symptoms raise dropout rates [21].

Careful weighting of machine learning algorithms and appropriate patient stratification can ease these problems and prevent harmful feedback loops [12,16,50]. Useful techniques include uncertainty calibration by subgroup and adversarial debiasing, which helps disentangle protected characteristics from latent risk factors [23]. In practice, this means adding demographic variables to models, using group normalisation terms, and evaluating performance continuously during development [23]. Recruiting participants from varied backgrounds, including those with special needs, and drawing on complementary and low-resource data sources will matter [10,21,30]. Taking socioeconomic and environmental influences into account during assessment can sharpen understanding of how symptoms present across groups and how presentation relates to underlying biological risk [11,27,45]. In our reading, these measures should not be treated as optional refinements applied after a model underperforms. Representative sampling and subgroup calibration belong at the design stage, as we argue in Section 4.2, since a field that frames equity as a goal cannot meet it by correction alone.

#### 3.2.2. Inequalities in Access and Limitations in Precision, Validity and Generalisability

The literature stresses that equitable access to emerging mental health models cannot be taken for granted [12,14,20,27,30,31,44,45,54]. Clinical research often draws on patients with mild or moderate symptom severity, which limits how well the resulting models apply to people with severe psychopathology [31]. Patients with Major Depressive Disorder or Schizophrenia, or those with social withdrawal and behavioural disorganisation, may opt out of precision psychiatry altogether. That reduces their access to new therapeutic options and widens the gap between patients with severe conditions and those with milder disorders such as anxiety disorders [32].

Economic inequality is another exclusion mechanism. Groups that lack the technological resources needed for precision psychiatry services, including genomic sequencing, advanced neuroimaging, and neuromodulation, can be cut off from the benefits [14,20,26,27,47,55]. Similar problems arise when behavioural data or other clinical variables are collected through AI or smartphones in what is called digital phenotyping [20,30,31].

Ethnic and racial minorities often have poorer access to mental health care and face worse prognoses [10], independently of disorder severity [55]. The same disadvantage affects minors from underserved communities, including African American and Latino populations [40]. Bias baked into psychiatric models, and systemic racism more broadly, can lock inequality in place by producing classifications that do not line up with the realities of protected groups [10].

A poor grasp of how predictive factors connect to outcomes deepens treatment disparities, since observed correlations may reflect sociopolitical bias rather than real clinical need [55]. Global health equity remains an ethical goal and one that matters particularly in psychiatry [20,28,30].

Predictive accuracy has not kept pace with the growth in available data, and the gap raises ethical concerns [5,12]. The risks of intervening have to be weighed against those of not intervening [26], and agreement is needed on what error rates can be accepted [10].

Database structure, size, and quality come under regular scrutiny, and their variety and scale make it hard to build coherent models that reliably predict psychiatric outcomes [6,16]. Integrating different kinds of biological data, including genomics, transcriptomics, proteomics, metabolomics, and microbiome information, is still difficult [16]. Model interpretation techniques can give inconsistent results when they are derived from low-quality data or used outside the contexts in which they were developed [11,19]. Some authors recommend validating AI-generated findings with conventional diagnostic methods to protect accuracy [19].

Attempts to simplify models for edge deployment, especially where data are scarce, can lower predictive accuracy [23]. Algorithm-driven changes in clinical practice can produce performative predictions that skew risk estimates after the intervention has happened [45]. This is why holdout sets, groups that are not exposed to the intervention, should be used with proper ethical safeguards [45].

AI approaches such as network-based algorithms and deep learning make it easier to handle multi-modal data and build large databases [16]. For precision psychiatry models, algorithms need to be tested in populations that reflect environmental and genetic diversity [4,6,10,11,33,34,51,54,56]. Generalisability improves modelling accuracy and helps address the subjectivity of psychopathology and the limitations of how mental health data are collected more generally [10].

#### 3.2.3. Potential for Misinterpretation

There are clear ethical worries about how predictive model outputs can be misread and about what follows from such misreading. Reductionist framings that put too much weight on biological, genetic, and pharmaceutical factors risk downplaying the environmental and psychological components of mental disorder onset, course, and persistence, as the biopsychosocial model reminds us. That imbalance can pull funding and attention away from psychosocial interventions and the data that support them, weakening a holistic approach [8,12,35,36,37]. Also, while precision psychiatry aims to separate mental disorders by genetic and pathophysiological features, researchers should not pursue precision-based work when clinical utility is lacking [8].

Specialist staff are advised to stay cautiously optimistic. Overstated benefits, without objective evaluation, create unrealistic expectations and erode long-term trust among patients and policymakers [12]. Bad prior experiences with precision medicine can also slow future uptake by shaping stakeholder attitudes in advance [15].

Uncritical reliance on algorithms raises concerns about both patient autonomy and medical judgement [37,57,58]. The danger becomes sharper when algorithmic errors can cause immediate harm and the capacity to challenge those recommendations has itself eroded [58]. Post hoc explanations and the illusion of thorough understanding can give clinicians more confidence than they have earned [23,56]. Clinicians should remain vigilant against cognitive biases [10].

#### 3.2.4. Explainability and Interpretability of AI

Insufficient explainability for clinicians, patients, families, and other stakeholders raises serious ethical concerns [4,49]. The black-box nature of many predictive models, which yield specific outputs without allowing for retrospective analysis, illustrates the point [12,19,23,25,33,34]. When intelligibility, accountability, and transparency are missing, clinicians struggle to justify their decisions, appropriate action becomes harder, and informed consent for therapeutic interventions is compromised. That in turn weakens trust in the wider use of AI and in its ethical integration into clinical care [6,8,12,16,19,33,36,39].

Conceptual problems also appear: limited understanding of biological mechanisms, difficulty in judging the clinical significance of genomic variables, and obstacles in translating data into useful clinical actions [6]. Algorithms with transparent internal structures, such as white-box analyses or Bayesian network models, can improve ethical acceptability by encoding causal or probabilistic relationships directly and by clarifying the size, prevalence, and direction of the factors involved [12,23,25]. Still, the flood of data produced by new technologies may exceed our capacity to understand how that information is generated [12].

Causal links matter. Mechanistic insight needs to be connected to new biological knowledge, pathophysiological pathways, and the phenomena that bear on healthcare decisions and health promotion [16,17,41]. Concepts such as multifinality, where the same factor leads to different outcomes depending on system structure, and equifinality, where different pathways lead to the same outcome, have to be kept in mind [58]. Efficiency can come at the cost of explainability [11]; deep learning often beats traditional methods on performance but loses interpretability, which signals the need to balance these priorities [10,11]. When no physiological explanation is offered for a diagnostic outcome, doubts about its authenticity tend to follow [41]. We take the view that this trade-off should be resolved differently in psychiatry than in fields with hard outcome labels. Because diagnosis here rests on subjective report, an opaque output cannot be checked against an objective standard, so interpretability carries clinical and not merely ethical weight, and white-box or causally informed models deserve preference at comparable performance, a position we develop in Section 4.2.

#### 3.2.5. Cost–Benefit Balance

The debate around precision psychiatry hinges on cost–benefit analysis, with particular attention to effectiveness and sustainability [9,10,12,31,34,35,54]. The technology is complex, implementation and operating costs are substantial, and healthcare infrastructure varies widely, which means heavy investment in equipment and training. These factors can bite hardest in resource-limited settings [26,33], and they sit against the backdrop of chronic underfunding in mental health research [12]. Adoption in clinical practice depends on evidence of superiority over conventional methods [12]. Some precision psychiatry tools may be less cost-effective in their early deployment [13,32]. Exceptions exist: pharmacogenetic polymorphism testing (agranulocytosis-clozapine, Stevens–Johnson-carbamazepine) [13,32] and stratified approaches to depression treatment [5] have shown promise. Beyond cost-effectiveness, attention is also needed for closer patient monitoring, more frequent consultations, and the extra time required to apply tools and interpret results [15,54].

Ongoing evaluation of potential risks and benefits matters, especially given the speed of technological change and the need to react to adverse outcomes [20,24,28,33,59]. Initial testing costs, utility, and risks of specialised interventions need to be weighed against long-term expenditures, disability-adjusted life years, and healthcare utilisation caused by poorly managed mental illness [10,46]. Emergency department visits, hospitalisation frequency, lost productivity, adverse reactions to interventions, and subjective patient experience should all inform this assessment [7]. Standardising advanced genomic data systems requires solid Information Technology (IT) infrastructure, skilled data management, and consistent access across providers [33]. AI-driven applications also have potential for matching health resources to individual patient needs [10].

#### 3.2.6. Reduced Perceived Benefit

Patients need to see that precision models produce real, measurable outcomes [15]. Nevertheless, it is certain that the current framework of precision psychiatry may not capture the subtle factors that rely on human interaction, which still drive much of clinical decision-making [15,50]. On the other side, the subjectivity of human judgement can miss aspects that sit outside current scientific paradigms [56].

Persistent biases against AI, worries about technological dependence, and concerns about the dehumanisation of care cannot be set aside [6,10,56]. Simulated therapeutic dialogues, for example, may lack the depth and nuance of genuine therapist interaction, which can undermine psychological integrity and user autonomy, and get in the way of a real interpersonal therapeutic relationship [27,59]. How these technologies are introduced and culturally absorbed therefore matters, since patients respond differently to continuous data monitoring and AI-guided therapy [24,34,47]. Social engagement, therapeutic alliance, and peer support cannot be delivered by algorithms alone, though this does not rule out AI as a supporting tool [27].

Clinicians may resist these methods because they see limited clinical benefit or worry about being compared and judged [52]. For new psychiatric models to take hold, evidence of their superiority must be available for the groups and individuals who have not yet benefited from traditional approaches: those with refractory conditions, atypical presentations, reported adverse treatment effects, high risk of treatment discontinuation, and families of people with mental disorders [6,15,28,30,52,54]. Patients at higher risk of poor outcomes should be matched to more intensive treatment strategies [5]. A drift toward neurocentrism can push attention away from non-medical interventions [41]. Social support, living environment, occupation, cultural context, and socioeconomic status still have to be integrated into the choice and nature of any intervention [30].

#### 3.2.7. Risk of Commercialisation

Premature commercialisation of predictive testing for mental illnesses, often driven by commercial interests, is an important ethical concern. The proliferation of mental health apps that lack reliability or patient utility is a clear example [12,27]. Generating revenue by sharing patient data with for-profit entities, even with consent, raises further questions (Crisis TextLine) [10]. Clinician reluctance to adopt new interventions sometimes comes from unease about endorsing commercially motivated products [43]. Decisions taken without professional advice can cause harm, feed misconceptions, and add to psychological distress and stigma [12]. Strong oversight is needed to prevent conflicts of interest, biased user information, undue pressure on clinical implementation, relaxed regulatory protections, and neglect of alternatives [12].

#### 3.2.8. Stigma and Response to Prognostic Information

Predictive models in precision psychiatry will identify people at high risk for developing a mental disorder. That early labelling, though informative, can also lead to stigmatisation, social prejudice, and self-stigma [12,17,30,32,34].

Some authors flag the misuse of genetic or personal data by third parties as a further barrier, particularly if employers or insurers gain access [15,26,27]. Such discrimination can cause economic harm, through denied employment or unfair refusal of insurance for individuals at risk [15,26,54]. The US Genetic Information Nondiscrimination Act of 2008 forbids this kind of data use [26].

Self-stigma has been linked to several negative effects for patients, including poorer emotional state, impaired social role functioning, and reluctance to seek medical help [12,54]. Some patients may withdraw from healthcare services altogether to avoid exposure [13]. The stigma of mental illness also reaches patients’ families [12,38]. Adverse social consequences have been noted in relation to ungrounded worries and lowered expectations for offspring who carry risk-related genes [15,38].

How patients respond to prognostic information has attracted a good deal of discussion. Knowing one’s risk for a mental disorder can encourage deterministic views of illness, and that in turn can undermine self-concept and well-being even before clinical manifestation, which may shape how the disorder unfolds [5,12]. Another issue is how prognostic information affects psychiatric diagnosis itself, given how much the diagnosis relies on retrospective patient reporting [12,56]. Disclosure of such data can shape what patients say, complicating the collection of the accurate histories clinicians need [12]. Access to screening tools outside clinical settings carries a risk of misinterpretation that can change help-seeking behaviour, undermine trust in treatment, and in some cases worsen psychopathology, including self-destructive behaviour [11,27].

#### 3.2.9. Informed Consent and Autonomy

Informed and ongoing consent is central to patient autonomy, freedom, and empowerment, and it is an ethical prerequisite for integrating new healthcare applications [16,17,25,33,54]. For patients to make decisions that reflect their values and preferences, without undue pressure, they need support and information on which they can rely, understand and process [30,32,35,43,48]. One challenge is deciding how much information to give, and of what kind, since predictive models produce probabilities that carry error, not certainties [10,12,37].

Information should be clear, transparent, and tailored to the patient’s level of understanding so that decisions and subsequent actions rest on solid ground [12,37,45]. Heavy medical jargon should be avoided, and the way information is conveyed should not become a tool of persuasion [15]. Building up patients’ digital literacy and engagement increases their confidence in making healthcare decisions [43].

Patients should be free to choose which results they wish to receive [45], with informed consent for the disclosure of findings that could affect life planning, clinical decisions, prognosis, or reproductive choices [12,17]. However, awareness alone may not offset the possible damage to self-image, quality of life, or mental health [12,38]. Information and consent for precision psychiatry assessments are ethically required, with an emphasis on prevention and risk reduction. Yet real complications arise in cases involving minors or individuals with cognitive impairment, who may not be able to give consent themselves [12,17]. These complications become sharper in preclinical stages, prodromal presentations, comorbid conditions, and early-onset severe mental disorders [12].

#### 3.2.10. Confidentiality

Confidentiality, privacy, and data protection come up again and again in the literature [6,11,13,16,18,19,27,31,32,33,34,35,45,47,52,56,60]. Genomic research and the data-sharing that surrounds it offer a clear example [6]. The large volumes and different types of information that feed precision psychiatry models, such as phenotypic, biological, and neuroimaging data, call for strict safeguards, respect for privacy, and accountability at both the collection and processing stages [17,18,20,32,47]. Unauthorised access to such data can harm patients, opening the door to discrimination or bullying, and disclosure can spill over to families [12,27,30,61].

Within the frame of cybersecurity, privacy, and confidentiality in these new models, recommendations converge on strong legal and scientific infrastructures for the secure collection, preservation, use, and communication of information, in line with local laws and with standards such as the Health Insurance Portability and Accountability Act and the General Data Protection Regulation [11,13,15,16,20,21,22,30,39,56,57]. Accountability for AI-generated outputs and patient privacy remain open problems [33]. The National Institutes of Health (NIH) runs clinical data repositories and uses procedures to improve researcher access while keeping information exchange under ethical governance [20]. Institutional Review Boards (IRBs) now increasingly require privacy and bias mitigation strategies as awareness of ethical data management has grown [23].

Encryption technologies, including homomorphic encryption, can protect sensitive neuroimaging and clinical data during analysis, and additional measures are needed to shield raw data from exposure [18]. Privacy can also be preserved through techniques such as adding noise to datasets, data fragmentation, and federated learning [21,23]. Cross-border database operation and governance need attention, as does the possibility of re-identification from supposedly anonymous datasets [27,39].

Another ethical problem sits between the confidentiality rights of people identified as high-risk and the informational rights of close relatives, particularly those who may share the genetic susceptibility and have a legitimate interest in knowing, so that they can act on it [12]. Loss of access to data or digital interventions after a study ends can itself put patients at risk [21]. Good methods should be paired with data protection policies that cover ownership, accountability, and data-sharing protocols [18]. Transparency and trust grow when patients receive feedback on how their data are used and keep a say in that use [23,34,57].

#### 3.2.11. Feasibility

Knowing one’s risk for a mental disorder without access to clinical or behavioural interventions that could reduce or delay it may discourage disclosure, lower the perceived value of testing, and in some cases harm wellbeing and quality of life [9,12,15]. The response is not universal, though. Some people still benefit from receiving the information even without a direct clinical advantage. Closer clinical follow-up, even when interventions are unavailable, can shorten the duration of untreated illness and limit deterioration or complications [12]. Access to preventive services after a risk assessment, through broader psychosocial intervention packages for patients and their families, can offer substantial benefits [12].

Greater patient engagement with precision psychiatry tools, from laboratory tests and neuroimaging to genotyping and electronic monitoring devices, should be encouraged [13]. In psychiatric settings this matters all the more, since patients can opt out of these tools because of their own psychopathology [13]. Adherence reinforced by collaborative engagement, information sharing, involvement in model development, and shared decision-making can improve the implementation and effectiveness of precision psychiatry [13,59].

#### 3.2.12. Education

Implementing precision medicine is hard without serious education and adaptation among all those involved [5,8,12,13,19,28,31,35,36,37,43,48]. Understanding of genetics, epigenetics, neuroimaging, and AI matters for patients and clinicians alike [16,36]. For physicians to use advanced techniques such as pharmacogenetics and pharmacogenomics well, they need a solid grasp of the concepts, trust in their utility, familiarity with application, and the ability to explain what these tools mean to patients and colleagues while keeping clear of conflicts of interest [12,13,34,35,36]. Scientific knowledge, good training, and strong communication skills are key [4,10].

Continued training in communication, interpretation of model outputs, statistics, information systems, genetics, and heritability of risk is needed [8,13,15,30,54]. Such training builds acceptance and confidence in new tools among physicians, and feedback from everyday experience deepens expertise over time [15,62]. Structured educational programmes at undergraduate and postgraduate level, continuing education conferences and seminars, and digital training resources can all support the effort [7,13,30]. Investment in education is important, because clinicians carry ultimate responsibility for clinical decisions whatever decision-support systems are in place [6]. No clinician can master every discipline, though. Collaborative approaches and consortia with reciprocal feedback are needed to connect scientific advancement with clinical practice [46,54]. The introduction of digital navigator roles within evolving health systems will help with the integration of AI [10].

#### 3.2.13. Counselling

Counselling rests on full patient education and support so that autonomy grows and individuals can manage risk factors and symptoms [12]. Psychiatric genetic counselling is expected to grow in importance, especially for predicting familial risk, and it may reach newer populations, including younger people [12]. Who should deliver this counselling is still an open question. Genetic counsellors bring specialised expertise, while clinicians often bring greater accessibility and a stronger therapeutic alliance, which build trust and safety [12]. Chatbots and conversational agents can also support psychoeducation and engagement, provided safeguards are in place to reduce the risk of misinformation [27].

#### 3.2.14. Screening and Intervention in Minors

Predictive models raise particular concerns when applied to minors. Diagnostic predictions carry real consequences for young people, especially during the neurodevelopmental period [44]. Parents want what is best for their children and try to prepare for their future, but awareness of risk and deterministic framings in precision psychiatry can harm a child’s mental health and self-concept [12,40].

It is not clear whether telling minors about these risks yields real benefits, or whether it is right to assume they can give informed consent for testing [12]. On the other hand, parents’ desire to protect their children, particularly when mental health disorders are involved, is understandable [12]. Predictive testing in minors looks justified when early interventions can reduce risk or prevent disorders that would surface in adulthood [12]. Such measures can also support greater autonomy later in life [26]. But autonomy develops gradually and differently for each child, which is why the minor’s own perspective must be taken into account [12]. A careful balance is needed when explaining psychiatric genetic predispositions, especially where the information may exceed what a minor can meaningfully process [40].

Ethical concerns sharpen when interventions involve minors. Safety is the first issue, along with harm minimisation, which includes the risk of long-term negative outcomes and developmental consequences [29]. Neurostimulation applied to a developing brain for the treatment of developmental disorders, for instance, can affect neurodevelopmental trajectories and neuroplasticity [29]. Genome interventions meant to prevent disorders in offspring raise complex issues [9]. Clinicians must also work through consent processes that are anything but simple. Addressing the ethical and practical sides of these interventions calls for interdisciplinary work among paediatricians, neuroscientists, data scientists, and policymakers [29].

#### 3.2.15. Prenatal Screening and Reproductive Choice

The ability to predict the onset of mental illness and identify high-risk phenotypic traits can influence reproductive decision-making [6]. Unlike physical illnesses, which are often seen as more serious or life-threatening, mental disorders are usually approached with more optimism about future treatment prospects. Routine prenatal screening for mental disorders and pregnancy termination based on adverse findings therefore find limited support, and resistance is grounded in concerns about stigmatisation and eugenic implications [12].

#### 3.2.16. Forensic Medicine and Forensic Psychiatry

Precision psychiatry also reaches specialised branches within psychiatry and other medical disciplines, including forensic medicine and forensic psychiatry. Precision-driven methods, such as improved therapeutic monitoring and genomically guided interventions, can support clinical practice by addressing long-standing challenges, including the prevention of suicidality and criminal behaviour [6,25]. However, introducing behavioural genetics into this field entails ethical costs. These include prioritising risk assessment over individualised treatment, shifting societal views of crime, and reinforcing social inequalities. The result can be harsher sentencing, reduced access to rehabilitation, and greater social exclusion of offenders [61]. The identification of biological correlates of behaviour and impulsivity raises a further question about how accountable individuals are for their actions and about the risk of premature attributions of guilt or of labelling people as inherently high-risk [41,61]. Relying on probabilistic genetic assessments in the justice system could erode the presumption of innocence and draw attention away from the psychological and socioeconomic factors that, if addressed, could help prevent crime and support rehabilitation [61]. Overstating predictive capability without sufficient scientific validation can produce ambiguous interpretations and unclear outcomes, with consequences for judicial decisions and a risk of linking severe mental disorders too strongly to crime and violence [25].

### 3.3. Need for Guidelines

Precision psychiatry needs clear operational standards and guidelines to direct the use of digital health technologies, biomarkers, AI, diagnostic tests and genomic analyses, and the management of clinical findings for both diagnosis and treatment. Consistent communication of principles and procedures to patients, and transparency in decision-making, matter just as much [19,20,31,42,43]. Current evidence suggests that gaps in the regulatory framework can lead to misinterpretation of results and to commercial interests taking precedence over clinical validity [12]. Reviews of Food and Drug Administration (FDA) approvals raise concerns about scientific rigour and point to a need for better quality across healthcare sectors [43]. Differences between computational models and human expertise also cannot be ignored [10]. Legal protections would give clinicians more confidence in using emerging medical technologies [10].

As new models of medicine are rolled out and novel diagnostic tests gain traction, early uncertainty about evolving technologies is to be expected [12]. Targeted frameworks for new care systems can ease concerns about the clinically, ethically, and legally sound use of genomic testing and neuroscience, and support wider acceptance [5,6]. Precision medicine aims at permanent or substantially improved treatment outcomes through interventions such as genetic editing and neuromodulation, and that raises fresh ethical questions. Informed consent, long-term effects, and the potential for misuse call for more detailed, context-specific guidelines [28].

For efficiency, clinical validity, and consistency across approaches, standardised protocols and licenced operating platforms matter, as do practitioner vigilance and cultural competence. These measures support adherence to the core ethical principles of beneficence, non-maleficence, autonomy, fairness, transparency, accuracy, trust, and professional competency [7,13,20,34,43,59]. Ethical review boards should oversee every part of machine learning model design, identify bias, assign accountability for AI-related errors, and encourage oversight by interdisciplinary teams [18,33,59]. Researchers should disclose how their systems are developed, who owns them, how they are trained, who is meant to benefit, what the business model is, and where the funding comes from [59].

## 4. Discussion

### 4.1. Key Insights

Clinicians, patients, their families, and future generations generally view the growth of risk prediction tools and the possibility of personalised interventions in a positive light. Acceptance is not universal, though. Perceptions of benefit and understanding of precision psychiatry depend on how information is communicated, how data are gathered, how stigma is approached, and whether meaningful intervention is actually possible. Risk assessment results must be conveyed in line with patient preferences and priorities, given the weight they may carry in a person’s life [43,48]. The same goes for initial consent to testing and for the autonomy patients retain over what follows. A clinician’s mix of social competence and scientific judgement shapes effective communication, accurate assessment of severity, and recommendations for appropriate intervention [2].

Positive views on precision psychiatry assessments tend to centre on improvements in quality of life and clinical outcomes, rather than on perceived risks, anxiety, or possible self-stigmatisation, which can provoke negative emotional responses and discourage positive change. In more severe mental disorders, several factors need weighing at once: the patient’s awareness of why participation matters, the cognitive capacity to understand how these models operate, social attitudes toward mental illness, and how stigmatisation can narrow the opportunities open to affected individuals.

Looking ahead, families may play a larger role in precision psychiatry by protecting members and reducing risk for younger relatives, with extensions that reach into prenatal screening [12]. Screening or intervention in minors calls for particular care, since this group has limited legal autonomy and decision-making capacity. Children’s own perspectives must be part of the picture. They should receive information suited to their ability to grasp risk, prevention, and the need for interventions that could improve current or future wellbeing [12,40]. Early intervention can affect future autonomy if consent is withdrawn later, but it can also support greater independence and better mental health outcomes over time.

A stronger emphasis on biological determinants alongside psychosocial factors in the aetiology and pathogenesis of mental disorders opens up complex questions about stigma [12,41]. Some argue that genetic, epigenetic, and neurophysiological explanations reduce critical attitudes and blame directed at patients. Others worry that deterministic readings of risk stir up fear and push affected individuals toward social exclusion. The emergence of a new category, those at risk of developing mental illness, raises questions about emotional responses, environmental interactions, and how screening results may themselves influence disease onset [12]. The interplay between perceived and biological risks runs through social behaviour, help-seeking, and treatment response. Stigma remains a real challenge that demands continued research across social, clinical, and psychological fields.

Risk assessment aims to prevent adverse outcomes by changing risk factors. Psychiatry at present does not have the precision needed to estimate the probability of a mental disorder with high reliability. Advances in therapeutic intervention may soften the weight of perceived risks as accuracy improves. Until then, scientific rigour must be maintained, and overstatement or oversimplification of new findings avoided. Adherence to ethical standards and independence from financial motives will be key to building trust around precision psychiatry. Serious engagement with bioethical questions lays the ground for gradual implementation, while continuous evaluation of the ethical and social implications is needed to minimise harm from delayed or misdirected care.

Future gains in predictive accuracy will likely depend on high-dimensional data. Severe psychiatric disorders are markedly heterogeneous, with multifactorial causes and varied presentations. Precision psychiatry is expected to lag behind other specialties because neurobiological frameworks remain incomplete and biomarker contributions are limited [2]. Validating findings within conventional care settings would improve credibility and support wider adoption. If the required accuracy proves out of reach, greater attention may turn to interpretive analysis of symptoms and patient categories rather than to mechanistic models. Sociodemographic variables are important in predictive modelling, so their interpretation and sources need careful checks. Representative sampling is needed for effective model design [2]. An overemphasis on biological and pharmacological aspects could also draw resources away from psychosocial interventions, possibly reflecting the limits or resource constraints of the latter [8,35,37,41]. Precision psychiatry may be emerging as a response to historically inadequate psychiatric treatment or as part of evolving policy toward better patient management.

Current industry and economic practices still push standardised treatments aimed at broad populations, with profitability and symptom management in the foreground [1]. Greater personalisation of treatment will be taken up only if it proves more cost-effective than and clinically superior to traditional methods, and that conclusion requires a thorough look at benefits, costs, and risks. Knowledge of adverse effects in specialised interventions is often limited, especially when the target populations are small. Predicting and managing mismatches, such as drugs unsuitable for a patient’s genotype, remains complex [1]. Economic realities pull in different directions: lower demand for specialised medications against the higher prices the industry needs to recover its investment [1]. Multimodal models bring higher costs and the possibility of unequal access [2]. An ever-growing number of targeted intervention models also risks inefficient resource use and market fragmentation [1,2]. Refining existing frameworks may be more productive than generating new ones without restraint. Equitable access to precision psychiatry cannot rest on profit alone; public funding will be needed so that socioeconomically disadvantaged groups and people with rare conditions are not left out [1]. But government and insurance policies, together with industry interests, can still slow innovation [1].

Technological advances, stronger statistical methods, and wider use of artificial intelligence have greatly increased data volume and analytical capacity. Even so, transparency about the underlying mechanisms remains limited, which holds back interpretability and explainability. The ethical principle of explainability points to clarity and accountability in AI-driven decisions, yet accuracy and bias reduction often take priority [22,36]. Medicine has long used interventions without full grasp of their biological mechanisms, supported by empirical evidence of efficacy [12]. That experience shaped theoretical work on the pathophysiology of mental disorders, though gaps in knowledge remain and motivate the search for more specialised and individualised interventions. Diagnosis still leans heavily on subjective patient reports and clinical examination, which is why objective markers, among them biomarkers, neuroimaging results, and genetic variants, are so urgently needed to improve recognition and diagnosis. That shift is expected to replace the long-standing trial-and-error approach with greater objectivity and precision [14].

Interdisciplinary collaboration is central to the development and implementation of new precision psychiatry models. Clear guidelines will give clinicians confidence in their use, and active oversight will help catch errors and biases early. AI takes on a growing support role, but clinicians still hold responsibility for communicating information, advising on decisions, and reviewing outcomes while honouring patient involvement and preferences as precision psychiatry evolves. Without strong clinician–patient communication and partnership, there is a real risk of reducing people to data points within Big Data analytics [14]. Continuous evaluation of how precision psychiatry models affect the therapeutic relationship will matter [2].

Precision psychiatry is a dynamic field that pulls together varied data sources, testing modalities, and intervention strategies. Focused work on particular components, among them machine learning, pharmacogenomics, genetic testing, gene therapy, and microbiome research, may yield deeper insight.

### 4.2. What This Review Adds

This narrative review contributes to the existing literature in three main ways. First, it moves beyond the conventional listing of ethical principles to focus on the practical tensions that arise when these principles conflict in real-world applications. Second, it maps these tensions across the full pipeline of precision psychiatry modalities, from data acquisition to clinical implementation. Third, and most importantly, it sets out the authors’ explicit positions on how these tensions might best be navigated. The four positions are developed below.

First, equity will not emerge passively from technological progress. Current trajectories risk widening rather than narrowing disparities, because individuals with severe mental illness, who stand to benefit most, are also those most likely to be excluded through symptom severity, social withdrawal, or limited digital access [31,32]. Representation and subgroup performance must therefore be treated as non-negotiable design requirements from the outset, rather than posthoc corrections. Public investment and targeted recruitment from underserved populations are essential if precision psychiatry is to honour its equity claims. In practice, this means reporting subgroup performance as a standard metric and treating a model that works only for the well-represented as unfinished rather than deployable.

Second, the accuracy-interpretability trade-off must be evaluated differently in psychiatry than in other medical fields. In disciplines with clear, objective endpoints, sacrificing transparency for predictive power may be justifiable. In psychiatry, where diagnosis and outcome assessment rely heavily on subjective experience and clinical judgement, opaque black-box models undermine both clinical safety and ethical accountability [10,11,41]. When performance is comparable, white-box or causally informed models should be prioritised. Where an opaque model is materially more accurate, its use should carry an explicit account of what cannot be explained and a defined route for the clinician to override it.

Third, the stigma associated with risk prediction is not a unidirectional phenomenon. Biological and genetic explanations can reduce self-blame while at the same time fostering deterministic thinking, lowered expectations, and self-stigmatisation, particularly within families and for at-risk minors [5,12,15,38]. The relevant question is therefore not whether prediction increases or decreases stigma overall but for whom, under which framing, and whether a meaningful intervention follows disclosure. In the absence of effective interventions, the ethical case for routine risk disclosure weakens substantially. Disclosure should therefore be tied to an actionable pathway and risk results that lead nowhere should be offered with caution rather than as a default.

Fourth, the promise of individualised care stands in tension with an industry that still profits from standardised products for broad markets. Greater personalisation will be adopted at scale only where it proves both clinically superior and cost-effective, and that judgement depends on transparent appraisal of benefits, costs, and risks. Public funding will therefore be needed so that socioeconomically disadvantaged groups and people with rare conditions are not left behind, since equitable access cannot rest on commercial incentives alone [1,2]. Cost-effectiveness against existing care, not novelty, should be the threshold for adoption, and tools that cannot clear it should not enter routine practice on the strength of their sophistication. These four positions are offered against an evidence base still dominated by reviews and conceptual work, with comparatively few original empirical studies, which is itself a reason an interpretive synthesis that states the tensions plainly is useful at this stage and a signal of where future empirical research is most needed. The domains corresponding to these cross-cutting touchpoints are summarized in Table 1.

### 4.3. Limitations

This narrative review is subject to several limitations that should be acknowledged. One limitation of our review was restriction to English-language articles, excluding some materials not freely available, although requests for access could have been made. Furthermore, the literature search was limited to two electronic databases (PubMed and Google Scholar), and therefore relevant studies indexed in other databases may not have been captured. In addition, the screening and selection process was primarily conducted by a single reviewer, which may increase the risk of selection bias, although consultation with a second reviewer was undertaken in cases of uncertainty. A formal quality appraisal of individual sources was not performed, consistent with the narrative design, so the synthesis should be read as an interpretive map of the field rather than as a graded assessment of evidence strength. Nonetheless, the predominance of review articles in recent publications (2020–2026) with international representation, enhances the relevance and generalizability of our findings.

## 5. Conclusions

This narrative review mapped the major ethical tensions that accompany the development and use of precision psychiatry and advanced four explicit positions to help navigate them. Equity must be treated as a design requirement rather than a hoped-for outcome. Interpretability carries special clinical weight in a discipline grounded in subjective experience. Stigma is bidirectional and should be judged by its framing and by the availability of actionable intervention. Personalised care must demonstrate tangible superiority over existing standardised methods before it can claim priority.

Precision psychiatry has the potential to reshape psychiatric practice and the therapeutic relationship itself. Realising that potential responsibly will require more than technological sophistication. It will demand sustained interdisciplinary collaboration, transparent guidelines, rigorous evaluation of both benefits and harms, and continuous ethical vigilance. Ultimately, the value of precision psychiatry will be measured not by the complexity of its models but by whether it delivers more accurate, more equitable, and more humane care than the approaches it seeks to replace.

## Figures and Tables

**Table 1:** Unresolved tensions in the ethics of precision psychiatry and the position taken in this review.

Domain	Unresolved Tension in the Current Literature	Position Taken in This Review
Bias and representation	Debiasing methods are described, yet rarely prioritised or built in at model inception [10,12,16,21,23,30].	Representation is a design requirement. Subgroup calibration and diverse recruitment belong at the start, not as a later correction [10,12,16,23,50].
Access and equity	Equity is named as a goal while severe-illness and low-resource exclusion persist [10,20,28,30,31,32].	Severity-related self-exclusion is structural in psychiatry and must be planned for, with funding for access, rather than assumed away [14,20,28,30,31,32].
Explainability	Accuracy is consistently favoured over interpretability [10,11,19,23,33,34].	Where diagnosis rests on subjective report, interpretability carries clinical weight [10,11,41]. White-box or causal models deserve preference at comparable performance [12,23,25].
Reductionism	The biopsychosocial model is affirmed, but biological framings dominate funding and attention [8,12,35,36,37].	Precision work should declare clinical utility at the outset and resist precision pursued for its own sake [12,23,37].
Cost and benefit	Few tools show cost-effectiveness in early deployment [13,32].	Evaluation should weigh long-term disability-adjusted life years and the cost of untreated illness, not only the upfront price [10,46].
Stigma and prognosis	Early labelling informs planning yet can harm self-concept before onset, and it cuts in two directions [5,12,15,30,38].	The question is not whether prediction rates raises or lowers stigma but for whom, in which framing, and whether an intervention follows [5,12,15,38].
Consent and autonomy	Probabilistic outputs complicate what informed consent can mean [10,12,37].	Consent should be ongoing and tiered to what the patient chooses to learn, supported by plain communication [12,15,43,45].
Privacy	Strong frameworks are cited, but re-identification risk remains real [21,23,27,39].	The confidentiality of high-risk individuals and relatives’ interest in knowing form a genuine conflict that needs explicit rules [12,18,34,57].
Vulnerable settings	Minors, prenatal screening, and forensic use are treated in isolation [6,12,25,29,40,41].	These settings concentrate the hardest trade-offs and warrant dedicated, interdisciplinary guidance [12,25,29,40,61].
Therapeutic relationship	AI is framed as support, while dehumanisation of care is feared [2,14,27].	Algorithms supplement but cannot replace the alliance. Their relational effect needs continuous evaluation [2,14].
Governance and education	Guidelines are called for, yet regulatory gaps remain [12,19,20,42,43].	Clinician responsibility is uncharged by decision support. Education and standards should precede wider roll-out [18,20,33,43,59].

## Data Availability

No new data were created or analyzed in this study. Data sharing is not applicable to this article.

## Supplementary Materials

The following supporting information can be downloaded at: https://www.mdpi.com/article/10.3390/jpm16070337/s1, Table S1: Distribution of the 62 included sources by type.

## Abbreviations

The following abbreviations are used in this manuscript:

AI	Artificial Intelligence
CYP2D6	Cytochrome P450 2D6
CYP2C19	Cytochrome P450 2C19
FDA	Food and Drug Administration
NIH	National Institutes of Health
IRB	Institutional Review Board
IT	Information Technology
