# Supplementary material for: A Narrative Review of Ethical Issues in Precision Psychiatry: Mapping Unresolved Tensions Across Modalities
Source: J Pers Med. 2026 Jun 23;16(7):337. doi: 10.3390/jpm16070337 (PMC13412752; doi:10.3390/jpm16070337)
Supplement: Supplementary file 1 [file jpm-16-00337-s001.zip › jpm-4344276-supplementary.pdf]

## Supplementary Material

*A Narrative Review of Ethical Issues in Precision Psychiatry: Mapping Unresolved Tensions Across Modalities*

**Table S1.** Distribution of the 62 included sources by type.

| Type of Source | IncludedStudies, n |
|---|---|
| Reviews and syntheses (narrative, systematic, scoping) | 29 [2,3,7,9,11–13,15–18,20–22,27,28,31–33,39,45,47–50,56,57,59,61] |
| Theoretical, conceptual, or commentary articles | 22 [4–6,8,10,14,19,26,29,30,34–36,42,44,46,51,52,55,58,60,62] |
| Originalempiricalstudies | 8 [1,23,24,37,38,40,43,53] |
| Doctoraltheses | 3 [25,41,54] |
| **Total** | **62** |

*Reference numbers correspond to the numbered reference list of the main manuscript.*
